# Healthcare and social needs of international migrants during the COVID-19 pandemic in Latin America: analysis of the Chilean case

**DOI:** 10.1177/17579759211067562

**Published:** 2022-03-20

**Authors:** Alice Blukacz, Báltica Cabieses, Edward Mezones-Holguín, José Manuel Cardona Arias

**Affiliations:** 1 Instituto de Ciencias e Innovación en Medicina, Facultad de Medicina Clínica Alemana, Universidad del Desarrollo, Las Condes, Región Metropolitana, Chile; 2 Centro de Excelencia en Investigaciones Económicas y Sociales en Salud, Universidad San Ignacio de Loyola (USIL), La Molina, Lima, Peru; 3 Epi-gnosis Solutions, Piura, Peru; 4 Innovations for Poverty Action, Ciudad de México, Mexico

**Keywords:** international migrants, social determinants of health, social vulnerability, Latin America, COVID-19

## Abstract

International migrants are a particularly vulnerable group in the context of the coronavirus disease 2019 (COVID-19) pandemic. Immigrants in Chile tend to experience multidimensional poverty and layers of social vulnerability. Our analysis aims to describe the perceived social and health-related needs of international migrants during the COVID-19 pandemic in Chile in terms of migration as a social determinant of health and layered social vulnerability. We carried out a qualitative analysis of responses to an open-ended question focused on the social and health-related needs linked to the pandemic included in an online questionnaire disseminated during April 2020 aimed at international migrants residing in Chile. The information gathered was thematically analysed. We included 1690 participants. They expressed needs related to health and others linked to the overall socio-economic and political response, employment, material conditions and psychosocial aspects. They also reported needs related to ‘being a migrant’. Additionally, some participants described situations of vulnerability. We analysed their needs and situations of vulnerability identified around the following emerging frames: (a) work and living conditions, (b) regularisation traps and perceived lack of support and (c) and physical and mental health needs. International migrants in Chile report experiencing interrelated layers of social vulnerability during the COVID-19 pandemic, where ‘being a migrant’ exacerbates physical and mental health risks. The issues revealed are immediate and direct public health challenges, as well as different aspects of social vulnerability linked to migratory status, employment and barriers to accessing healthcare that should be addressed through comprehensive policies and measures.

## Introduction

Estimating the social and health impact of the coronavirus disease 2019 (COVID-19) pandemic requires a comprehensive approach that involves considering the social determinants of health, among which migration is highly relevant. Migration is a transversal social determinant of health, insofar as individual health may be altered before, during and after the migration process and the circumstances of the individual migrant and their context changes (1,2). International migrants are defined as people who move away from their country of usual residence across an international border, temporarily or permanently, and for various reasons (3).

Beyond the direct threat to public health and individual health outcomes posed by the COVID-19 pandemic globally, indirect risks linked to social determinants of health and social vulnerability are becoming apparent in most countries (4). In this context, migrants and their families are often part of marginalised and vulnerable groups in societies and the challenges described in the emerging literature on COVID-19 and migration are multiple, including aspects related to the closure of borders (5), migratory status (6,7), informal or precarious employment as well as unemployment (8,9), lack of social protection, inadequate and overcrowded housing and lack of support networks and discrimination (10).

Vulnerability is a highly significant concept regarding health, and requires special attention in the context of the pandemic. Human vulnerability is an inherent, absolute and permanent characteristic or label, while social vulnerability is defined as constructed, dynamic, layered and modifiable (11). Connected to social vulnerability are social determinants of health, which can be structural, encompassing general socio-economic and political context, the socio-economic position and social class of an individual and their demographic characteristics, and intermediate, including the health system, as well as material circumstances, behaviour, biological aspects and psychosocial factors at the individual level (12).

Migration as a social determinant of health deserves a priority approach in the COVID-19 pandemic. Although the adversity experienced during the pandemic is not unique to the migrant population, and is not experienced equally among all subgroups of international migrants, it is crucial to identify their specific needs to promote adequate, timely and equitable solutions, as preventive and epidemic control approaches should include migrants as a vulnerable population (13).

Chile is a high-income country (14) receiving immigrants predominantly from Peru (27%), Venezuela (18%), Colombia (13%) and Haiti (11%). In 2019, the total number of foreign residents represented approximately 8% of the total population (1,492,522) (15). According to the 2017 National Socioeconomic Survey (CASEN, from the Spanish acronym), 24.6% of foreign-born residents experience multidimensional poverty four percent more than nationals, and 15.8% of the foreign-born population did not have any health insurance affiliation versus only 2.2% for nationals (16). International migrants in Chile face barriers to accessing healthcare, including migratory status, administrative issues, misinformation, costs and discrimination (17,18). Additionally, they may face exacerbated social and health needs compar-ed with nationals, considering their precarious transit conditions, entering the country through nonauthorised crossing-points and difficulties in obtaining temporary or permanent residency (18,19).

Considering that Latin America has become a hotspot of the COVID-19 pandemic (20) and that the region presents high levels of social and health inequality, assessing the situation of international migrants in Chile in the context of the pandemic is of high interest on the global health agenda. The research question guiding our analysis is: what were the perceived social and health-related needs of international migrants facing the COVID-19 pandemic in Chile regarding migration as a social determinant of health and layered social vulner-ability? The objective of the analysis is to identify self-perceived dimensions of social and health vulnerability experienced by international migrants in Chile during the COVID-19 pandemic, with the aim of contributing to the generation of primary evidence around migration as a social determinant of health and the related layers of social vulnerability in the context of the pandemic.

## Methods

### Study design

We carried out a qualitative thematic analysis of written responses to an open-ended question nested within a larger cross-sectional quantitative study with an opinion poll design, which sought to identify (a) the level of knowledge that immigrant populations in Chile had around COVID-19 and prevention measures and (b) their immediate needs and concerns towards the future as a consequence of the pandemic.

An online questionnaire was available for completion via Google Forms, which is easy to access from any device, between 4 April and 24 April 2020, in Spanish and Haitian Creole.

The questionnaire was based on previous studies about migration and health in Chile and population surveys including migrants. It included 31 multiple-choice questions organised in the following sections: (a) sociodemographic data, (b) migratory process, (c) living conditions, (d) knowledge of COVID-19 based on WHO official information, (e) coping strategies, (f) compliance with prevention measures recommended and implemented in Chile at that time (April 2020), (g) understanding and opinions on the information available regarding the pandemic in Chile at that time.

In addition to these questions, we included an open-ended question, allowing for a qualitative analysis, which is the focus of this paper:

- What do you need to feel calm despite the issue we are currently facing because of COVID-19? (Translated from Spanish)

The original question in Spanish was as follows: *¿Qué necesita para sentirse tranquilo con este problema que enfrentamos hoy de COVID-19?* Its version in Creole was the following: *Kisa ou bezwen pou ou santi’ou trankil ak pwoblèm nap travèse jodia ak KOVID 19?*

All participants (*N* = 1690) answered this question. The question had no word count limit for responses, ranging from one word to several short sentences.

The questionnaire was designed and pilot-tested by one of the authors (BC) with migration experts of partner institutions and eight international migrants from Venezuela, Peru, Colombia and Haiti to assess whether the questions were clear, understandable and culturally relevant. In addition, two multiple-choice questions were modified in order to improve clarity.

### Recruitment of participants and data collection

By a nonprobabilistic sampling, participants were recruited by disseminating the questionnaire among social networks, partner organisations and the public healthcare network to reach individuals self-identifying as international migrants residing temporarily or permanently in Chile at the time of the questionnaire. Access to the internet from any device and over 18 years old were the only selection criteria.

### Data analysis

The data collected for the selected open question was stored in a Microsoft Excel spreadsheet (Microsoft Corporation, CA, USA) to facilitate the analysis carried out by one author (AB). The first step was a general reading of all the answers to map out broad thematic categories, following the concept-indicator model in open coding or constant comparison of regularly occurring textual material (21). In this process, we identified a proportion of the respondents not directly answering the question, taking the opportunity of an open-ended question to describe the situation they were facing at the time rather than explicitly describing their needs. The second step was making the decision, by two of the researchers (BC and AB), to carry out two processes of codification, one for each type of answer: needs and description of the situation. The third step was performing an inductive coding process, whereby we formulated a scheme of categories and codes and refined it iteratively as the data was reviewed (22). Finally, we defined saturation when no new code emerged from the data (23,24). The fourth step was organising the results in tables by categories, codes and respective supporting quotes.

### Ethics

Data were collected voluntarily and entirely anonymously, with no way of identifying the participants. Participants gave their informed consent by ticking the corresponding box before accessing the questions. We informed participants of the purpose of the study, explaining that their participation would contribute to knowing the impact that COVID-19 could have on the population to develop prevention and intervention programmes. Additionally, we notified them that participation entailed completing an online questionnaire, that data would remain strictly confidential and anonymous, and that the collected data were for research purposes. Furthermore, we introduced the entities conducting the survey. Finally, we did not explore sensitive information, such as migratory status, whether they intended to stay in the country and how long, self-perceived discrimination, whether they worked formally or informally, living conditions or income.

## Results

### Sample description

Of the 1690 total respondents, 67% self-identified as female, 33% male and less than 0.1% other. The average age of the total sample (*N* = 1690) is 38 years, with a standard deviation of 9.84. Most of the respondents were from Venezuela (59.70%), Colombia (13.55%), Haiti (5.38%) and Peru (4.38%), among 35 other countries. Most participants (62.8%) had been in Chile for 1–5 years at the time of response, 16.8% for 6 months to a year, 8.9% for 5–10 years, 7.9% over 10 years and 3.3% for less than 6 months.

Among the respondents, 74% reported university-level education, 24% secondary-level and 2% primary-level. Regarding labour status, 58.5% reported working in either the formal or informal sector, 40.1% reported not being employed but wanting to work and 1.3% reported not working and not wanting to.

Finally, 62.7% reported using public health insurance, 18.5% had no health insurance, 15.4% used private health insurance, 2.4% did not know their health insurance status and 0.8% reported using another type of coverage.

### Frames of analysis

**Figure 1.**
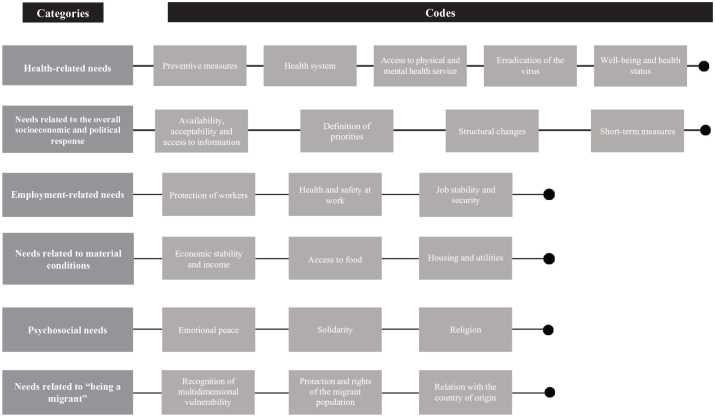
Emerging categories and codes related to expressed needs.

**Figure 2.**
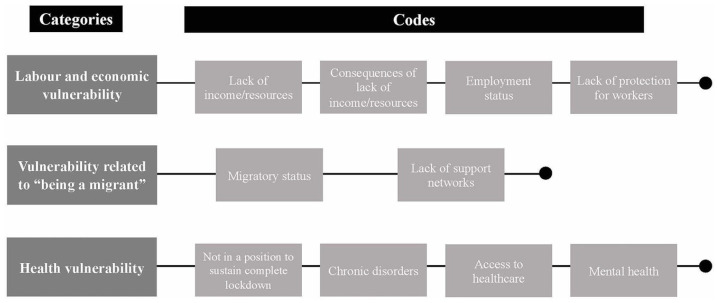
Emerging categories and codes related to described situations.

On the one hand, the participants expressed a diverse range of needs, some of which were directly health-related (Figure 1). Moreover, several participants took the opportunity of an open-ended question to write about the situation they were facing (Figure 2). Additionally, a small proportion (*n* = 13) of participants reported not needing anything.

We identified three frames of analysis:

- Work and living conditions: consequences of precarious labour and economic instability;
- Regularisation traps and perceived lack of support to international migrants;
- Physical and mental health needs: access to care and prevention.

For each frame, we described the main results and presented selected supporting data.

### Work and living conditions: consequences of precarious labour and economic vulnerability

The participants reported needs around employment, such as health and safety measures at work and measures to protect the job market in general and maintain wages. Others expressed the need to maintain their job or to find one. Additionnally, when describing their situation, some participants mentioned losing their job or not finding one, and the lack of social protection for informal and independent workers. For instance, a participant expressed the need for guaranteed job security in case she were to fall ill:

‘(I need) protection for workers, meaning, if I get COVID-19, still receive my salary and not lose my job after.’

Material conditions were often brought up with employment, and needs focused on economic stability and income. Additionally, needs around income or reported loss of income were associated with food security, housing, utilities and affordable medicines. In this context, some participants mentioned specific measures aimed at protecting small and medium enterprises as well as households; for instance, measures linked to social protection, freezing rent and utility payments or actively preventing evictions:

‘(I need) a law to cancel payments for rent and utilities for at least 3 months in order to make sure we can buy food with the little money we make. It would also prevent potential evictions for failing to pay rent. Unfortunately, this would make the situation worse; it is something we cannot control.’

### Regularisation traps and perceived lack of support to international migrants

Needs and vulnerability linked to work were also brought up with relation to migration, especially the length of processes to obtain or renew residence permits, leading to difficulties finding a job or falling into the informal market:

‘(I need) support for immigrants who do not have their ID yet or whose (residence) application is still not being processed, who have worked informally, because it seems like we are invisible to everyone.’

With regard to material conditions and migration, the participants mentioned migratory status as well as lack of network and lack of attention towards migrant communities and the strain that they may be experiencing:

‘(I need) support to be able to pay rent at least, because, if we get evicted, we cannot rely on being able to stay with relatives.’

Conversely, needs related to employment and economic stability were mentioned with relation to being able to pay for residency permits:

‘(I need) job stability (. . .) to be able to proceed with the application for the permanent residence permit.’

### Physical and mental health needs: access to care and prevention

Many of the needs expressed regarding health referred to increasing prevention measures, including mandatory lockdowns, accessible and affordable protective equipment or promoting collective responsibility and compliance to prevention measures. Moreover, in some cases, these needs were mentioned together with income to sustain a lockdown or buy protective equipment, health and safety at work or general living conditions including overcrowding:

‘I wish this country could be put under lockdown, complete lockdown, because it is useless for some of us to be quarantined while others are not, at least in my house there are 11 rented rooms, and I am careful and stay home, but everyone else is going out to work and they work with customers, and this is stressing me out as I have a chronic illness.’

In terms of health-related needs and situations of vulnerability linked to migration, access to healthcare was mentioned together with migratory status, lack of knowledge on the Chilean healthcare system or the need to receive further information and general support:

‘(I need) the guarantee that we will all receive good healthcare no matter our economic or migratory status if we get infected.’

‘I do not know how to use the healthcare system, and the only time I tried to use the emergency services at the hospital, they did not even measure my blood pressure; in fact, they made me feel bad for going because “I was not dying”. I am scared that something like this could happen again in an even more precarious situation than that one.’

Participants also mentioned that they needed the virus to disappear entirely or partially for their peace of mind. This situation can link to psychosocial needs, where participants report needing to feel calm, secure, safe, or have certainty that things will turn out well. In addition, they mentioned needing mental health support, with participants experiencing stress and anxiety. Furthermore, certain needs related to the country of origin – such as wishing to return, contacting relatives, and ensuring their wellbeing – also were expressed:

‘All I need is to make sure my parents, who are in Venezuela, stay well (they are over 60).’

## Discussion

Our findings suggest that the health and social needs of international immigrants facing the COVID-19 pandemic in Chile are diverse, and reveal different and often interrelated layers of social vulnerability. Migration as a social determinant of physical and mental health is a valuable concept for analysing interrelated layered social vulnerability, considering the social factors that affect health and operate to either include or exclude individuals and communities from adequate healthcare and the resources and experiences that foster health (25). Migrants are often in situations of poverty and social exclusion, working in hazardous environments, and experience poor living conditions, which affect their overall health and wellbeing, as well as experiencing barriers to accessing healthcare and social services (1). This situation is deeply worrying in the context of efforts to achieve universal health coverage and in the context of a global pandemic.

Although some of the instances of vulnerability and needs expressed can reasonably be assumed to be shared by at least a proportion of the local population, such as economic hardship, loss of income and employment, marginalisation and lack of institutional support, we found that a dimension of social vulnerability experienced by international migrants seems to stem from two main aspects of ‘being a migrant’: migratory status, which leads to ‘regularisation traps’, and lack of social support and networks. In the context of the pandemic, a precarious migratory status brought an additional layer of vulnerability linked to limited access to formal support as well as uncertainty surrounding whether the pandemic would slow down residence application processes. The impact of the pandemic on processes of migratory regularisation and subsequent access to social rights was also observed in other countries (26). Additionally, although not explicitly mentioned by the participants, being an international migrant in Chile implies facing structural racism and xenophobia (27) leading to exacerbated social vulnerability and may determine adequate access to critical services such as healthcare.

Regarding healthcare needs, participants express-ed uncertainty around access to health services and the need to receive clear instructions on what to do if they fall ill, while reporting not knowing how to navigate the healthcare system. Both results are consistent with previous studies on barriers experienced by migrants to accessing healthcare at the global level (28–30) and in Chile (17,31,32), where the introduction of Decree no. 67 aimed at granting access to public healthcare to international immigrants without an income regardless of their migratory status (33), mitigated administrative barriers to healthcare (34). During the pandemic, similar barriers were identified at the global level (35–37). These needs and concerns call for increased attention to guarantee equitable access to healthcare, especially during the pandemic, as increased demand for healthcare may further exclude vulnerable populations like international migrants.

Additionally, the reported lack of support networks in Chile exacerbated feelings of vulnerability. Although transnational ties usually constitute a source of social support (38), being away from relatives and friends might constitute a source of increased anxiety in the context of the pandemic. Participants expressed needs related to mental health, sometimes in connection with uncertainty related to employment, income, and housing. These findings are highly relevant both in the immediate context of the pandemic and in the longer term, as the adaptation of mental health services is needed, especially for vulnerable populations (39).

Migratory status and adversity perceived as stemming from ‘being an international migrant’ was also mentioned regarding employment and income. In some cases, migratory status conditioned income or formal employment, and migratory status was conditioned by income or formal employment to pay for the administrative fees for the regularisation process or considering that a range of residence permits in Chile are conditional to employment (40). The needs expressed surrounding economic and job stability point to the social vulnerability of the respondents in the face of the adversity brought by the pandemic (41), which can be further exacerbated by migratory status. In turn, participants reported working in conditions that expose them to potential infection with the virus and reported barriers to health preservation specific to the pandemic context and related to income, such as not being able to afford protective equipment or staying at home.

At the national level, these findings show that international immigrants, albeit a diverse group, are particularly at risk, regarding health and other aspects of their life, in the context of a major crisis. In that sense, in Chile and other countries, the government must guarantee effective and adequate access to culturally appropriate physical and mental healthcare and make prevention measures, including sustaining lockdowns, accessible and not conditional to income or risks of losing a job. These dimensions intertwine with migratory status and lack of social support and networks as sources of social vulnerability and potentially higher physical and mental health risks.

## Conclusions

The perceived social and health-related needs of international migrants facing the COVID-19 pandemic in Chile reveal different and interrelated aspects of social vulnerability. Importantly, aspects of being an international migrant such as migratory status and lack of social support and networks may exacerbate already precarious employment condi-tions, living conditions or regularity of income, which, in turn, can affect their health outcomes and limit their capacity to respond to the adverse conditions emerging from the pandemic.

In that sense, the following recommendations can be made: (a) during the pandemic, migratory regularisation processes should be facilitated and streamlined, (b) institutional and civil-society-led initiatives to provide social support to isolated and marginalised international migrants should be set up, (c) requirements to receive government monetary and food assistance should not include migratory status, (d) the right to access healthcare regardless of migratory status and country of origin should be explicitly guaranteed and reaffirmed.

Our analysis presents some limitations. First, convenience sampling led to women and university-educated international people being predominant participants. Second, internet access was an exclusionary condition meaning that the question-naire did not reach the most vulnerable communities of international migrants in Chile, potentially leaving out other needs and aspects of social vulnerability from the analysis. Third, the analysis did not consider respondent subgroups by gender, country of origin or first language, which could have led to a deeper understanding of the needs reported. Finally, we did not distinguish the area of residence of the participants, according to which the needs expressed could have varied.

Despite these limitations, our results may inform the potential health and social risks for other population groups experiencing different forms of social vulnerability in Chile and Latin America, such as indigenous populations, people experiencing higher multidimensional poverty rates, and people in prison or detention (42). The findings are also relevant for policymakers and practitioners globally, where migration containment policies threaten response to COVID-19 (43). Addressing the health of migrants should be addressed equitably and comprehensively in the context of the pandemic (44) and other crises such as climate change. Finally, our results can serve as a basis for future research in migration and social dimensions of the COVID-19 pandemic.

## References

[1] Davies A Basten A Frattini C . Migration: a social determinant of the health of migrants. Eurohealth. 2010; 16: 10–12.

[2] Orcutt M Spiegel P Kumar B Abubakar I Clark J Horton R , et al. Lancet Migration: global collaboration to advance migration health. Lancet. 2020; 395: 317–319. 32007150 10.1016/S0140-6736(20)30107-0

[3] International Organization for Migration. Who is a migrant? [Internet]. International Organization for Migration. 2016 [cited 2020 June 29]. Available from: https://www.iom.int/who-is-a-migrant

[4] Abrams EM Szefler SJ . COVID-19 and the impact of social determinants of health. Lancet Respir Med. 2020; 8: 659–661. 32437646 10.1016/S2213-2600(20)30234-4 PMC7234789

[5] Fernández-Niño JA Cubillos-Novella A Bojórquez I Rodríguez M . Recommendations for the response against COVID-19 in migratory contexts under a closed border: the case of Colombia. Biomédica [Internet]. 2020 [cited 2020 April 29]; 40(Suppl 2): 68–72. Available from: https://revistabiomedica.org/index.php/biomedica/article/view/5512 10.7705/biomedica.5512 PMC7676824 33152189

[6] Page KR Venkataramani M Beyrer C Polk S . Undocumented U.S. immigrants and Covid-19. N Engl J Med. 2020; 382: e62. 32220207 10.1056/NEJMp2005953

[7] Bhopal R . Covid-19: undocumented migrants are probably at greatest risk. BMJ [Internet]. 2020 [cited 2020 May 12]; 369. Available from: https://www.bmj.com/content/369/bmj.m1673 10.1136/bmj.m1673 32345590

[8] Liem A Wang C Wariyanti Y Latkin CA Hall BJ . The neglected health of international migrant workers in the COVID-19 epidemic. Lancet Psychiatry. 2020; 7: e20. 32085842 10.1016/S2215-0366(20)30076-6 PMC7129812

[9] Koh D . Migrant workers and COVID-19. Occup Environ Med. 2020; 77: 634–636. 32513832 10.1136/oemed-2020-106626 PMC7476302

[10] Devakumar D Shannon G Bhopal SS Abubakar I . Racism and discrimination in COVID-19 responses. Lancet. 2020; 395: 1194. 10.1016/S0140-6736(20)30792-3 PMC7146645 32246915

[11] Cabieses B Obach A . Explorando la relación entre migración internacional, vulnerabilidad social y salud. Cuad Méd Soc. 2018; 58: 109–119.

[12] World Health Organization. A conceptual framework for action on the social determinants of health: debates, policy & practice, case studies [Internet]. Geneva: World Health Organization; 2010 [cited 2020 May 28]. Available from: http://apps.who.int/iris/bitstream/10665/44489/1/9789241500852_eng.pdf

[13] Brandenberger J Baauw A Kruse A Ritz N . The global COVID-19 response must include refugees and migrants. Swiss Med Wkly [Internet]. 2020 [cited 2020 May 12]; 150. Available from: https://smw.ch/article/doi/smw.2020.20263 10.4414/smw.2020.20263 32343357

[14] The World Bank. High income. Data [Internet]. [cited 2020 Aug 4]. Available from: https://data.worldbank.org/income-level/high-income

[15] Estadísticas Migratorias [Internet]. Departamento de Extranjería y Migración [Internet]. 2021 [cited 2020 April 21]. Available from: https://www.extranjeria.gob.cl/estadisticas-migratorias/

[16] Inmigrantes. Síntesis de resultados. CASEN 2017 [Internet]. Ministerio de Desarrollo Social; 2017 [cited 2020 April 21]. Available from: http://observatorio.ministeriodesarrollosocial.gob.cl/encuesta-casen-2017

[17] Cabieses B Chepo M Obach A Espinoza M . Towards universal coverage for international migrants in Chile: accessibility and acceptability indicators from a multi-methods study. Med Res Arch [Internet]. 2019 [cited 2020 Apr 22]; 7. Available from: https://journals.ke-i.org/mra/article/view/1889

[18] Cabieses B Obach A Blukacz A Vicuña JT Carreño A Stefoni C , et al. Vulnerabilidades y recursos de comunidades migrantes internacionales en Chile para enfrentar la pandemia SARS-CoV-2: Construyendo estrategias diferenciadas desde la interculturalidad [Internet]. Santiago, Chile: Universidad del Desarrollo; 2021 [cited 2021 Oct 10]; p.206. Available from: https://repositorio.udd.cl/handle/11447/3842

[19] Cabieses B Obach A Blukacz A Carreño A Larenas D Mompoint E . Migrantes Internacionales en Residencias Sanitarias en Chile durante la Pandemia COVID-19: Hacia una Respuesta Ética en Emergencias Sanitarias [Internet]. Santiago, Chile: Organización Mundial de la Salud; 2021 [cited 2021 Oct 10], p. 183. Available from: https://repositorio.udd.cl/bitstream/handle/11447/3848/Migrantes%20internacionales%20en%20residencias%20sanitarias.pdf?sequence=4

[20] Dyer O . Covid-19 hot spots appear across Latin America. BMJ [Internet]. 2020 [cited 2020 August 4]; 369. Available from: https://www.bmj.com/content/369/bmj.m2182 10.1136/bmj.m2182 32482681

[21] Williams M Moser T . The art of coding and thematic exploration in qualitative research. Int Manag Rev. 2019; 15: 45–55.

[22] Pope C Ziebland S Mays N . Analysing qualitative data. BMJ. 2000; 320: 114–116. 10625273 10.1136/bmj.320.7227.114 PMC1117368

[23] Birks M Mills J . Grounded Theory: A Practical Guide. London: SAGE; 2015.

[24] Urquhart C . Grounded Theory for Qualitative Research: A Practical Guide. Thousand Oaks, CA: SAGE; 2012.

[25] Castañeda H Holmes SM Madrigal DS Young M-ED Beyeler N Quesada J . Immigration as a social determinant of health. Annu Rev Public Health. 2015; 36: 375–392. 25494053 10.1146/annurev-publhealth-032013-182419

[26] Gautier L Poveda J-D Wakap SN Bouchon M Quesnel-Vallée A . Adapting care provision and advocating for unprotected unaccompanied minors in Paris in the context of COVID-19. Glob Health Promot [Internet]. 2021 [cited 2021 Jun 11]. Available from: https://journals.sagepub.com/eprint/DFMPTEYAWKSRCZCGCTHI/full 10.1177/1757975920984193 33438481

[27] Tijoux ME . Racismo en Chile: La piel como marca de la inmigración. Editorial Universitaria de Chile; 2016, 265 p.

[28] Yu M Kelley AT Morgan AU Duong A Mahajan A Gipson JD . Challenges for adult undocumented immigrants in accessing primary care: a qualitative study of health care workers in Los Angeles County. Health Equity. 2020; 4: 366–374. 32923841 10.1089/heq.2020.0036 PMC7484891

[29] Arrieta JDR . Banca, salud y estímulo del empleo: servicios públicos desde la óptica de los refugiados y solicitantes en Costa Rica. Rev Cienc Soc. 2017; 1: 111–129.

[30] Woodgate RL Busolo DS Crockett M Dean RA Amaladas MR Plourde PJ . A qualitative study on African immigrant and refugee families’ experiences of accessing primary health care services in Manitoba, Canada: it’s not easy! Int J Equity Health. 2017; 16: 5. 10.1186/s12939-016-0510-x PMC5223444 28068998

[31] Bernales M Cabieses B McIntyre AM Chepo M . Desafíos en la atención sanitaria de migrantes internacionales en Chile. Rev Peru Med Exp Salud Pública. 2017; 34: 167–175. 29177373 10.17843/rpmesp.2017.342.2510

[32] Cabieses B Oyarte M . Health access to immigrants: identifying gaps for social protection in health. Rev Saúde Pública. 2020; 54: 20. 32074219 10.11606/S1518-8787.2020054001501 PMC7017981

[33] Ministerio de Salud. Salud del Inmigrante [Internet]. 2019 [cited 2020 Apr 22]. Available from: https://www.minsal.cl/salud-del-inmigrante/

[34] Blukacz A Cabieses B Markkula N . Inequities in mental health and mental healthcare between international immigrants and locals in Chile: a narrative review. Int J Equity Health. 2020; 19: 197. 33148258 10.1186/s12939-020-01312-2 PMC7640394

[35] Hill J Rodriguez DX McDaniel PN . Immigration status as a health care barrier in the USA during COVID-19. J Migr Health. 2021; 4: 100036. 33778797 10.1016/j.jmh.2021.100036 PMC7979269

[36] Özvarış ŞB Kayı İ Mardin D Sakarya S Ekzayez A Meagher K , et al. COVID-19 barriers and response strategies for refugees and undocumented migrants in Turkey. J Migr Health. 2020; 1–2: 100012. 10.1016/j.jmh.2020.100012 PMC8352003 34405167

[37] Maldonado BMN Collins J Blundell HJ Singh L . Engaging the vulnerable: a rapid review of public health communication aimed at migrants during the COVID-19 pandemic in Europe. J Migr Health. 2020; 1–2: 100004. 10.1016/j.jmh.2020.100004 PMC7661962 33447830

[38] Herz A . Relational constitution of social support in migrants’ transnational personal communities. Soc Netw. 2015; 40: 64–74.

[39] Moreno C Wykes T Galderisi S Nordentoft M Crossley N Jones N , et al. How mental health care should change as a consequence of the COVID-19 pandemic. Lancet Psychiatry. 2020; 7: 813–824. 32682460 10.1016/S2215-0366(20)30307-2 PMC7365642

[40] Thayer Correa LE . La política migratoria en Chile en la disputa por los Derechos Humanos. An Univ Chile. 2019; 16: 15–26.

[41] Bojorquez I Cabieses B Arósquipa C Arroyo J Novella AC Knipper M , et al. Migration and health in Latin America during the COVID-19 pandemic and beyond. Lancet. 2021; 397: 1243–1245. 33812478 10.1016/S0140-6736(21)00629-2 PMC9753767

[42] Mesa Vieira C Franco OH Gómez Restrepo C Abel T . COVID-19: the forgotten priorities of the pandemic. Maturitas. 2020; 136: 38–41. 32386664 10.1016/j.maturitas.2020.04.004 PMC7195319

[43] Hargreaves S Kumar BN McKee M Jones L Veizis A . Europe’s migrant containment policies threaten the response to covid-19. BMJ [Internet]. 2020 [cited 2020 May 12]; 368. Available from: https://www.bmj.com/content/368/bmj.m1213 10.1136/bmj.m1213 32217531

[44] United Nations Network on Migration. COVID-19 does not discriminate, nor should our response [Internet]. 2020 [cited 2020 Jun 29]. Available from: https://migrationnetwork.un.org/covid-19

